# Biological mechanisms discriminating growth rate and adult body weight phenotypes in two Chinese indigenous chicken breeds

**DOI:** 10.1186/s12864-017-3845-9

**Published:** 2017-06-20

**Authors:** Tengfei Dou, Sumei Zhao, Hua Rong, Dahai Gu, Qihua Li, Ying Huang, Zhiqiang Xu, Xiaohui Chu, Linli Tao, Lixian Liu, Changrong Ge, Marinus F.W. te Pas, Junjing Jia

**Affiliations:** 1 Yunnan Provincial Key Laboratory of Animal Nutrition and Feed, Yunnan Agricultural University, Kunming, 650201 Yunnan Province People’s Republic of China; 2 Department of Food Science, Yunnan Agricultural University, Kunming, 650201 Yunnan Province People’s Republic of China; 3 Animal Breeding and Genomics Centre, Wageningen UR Livestock Research, Building 107, Radix, Droevendaalsesteeg 1, P.O. Box 338, 6708 PB, 6700 AH Wageningen, The Netherlands; 4 Yunnan Agricultural University, Kunming, 650201 Yunnan Province People’s Republic of China; 5 Dali University, Dali, People’s Republic of China

**Keywords:** Growth rate, Chicken (*Gallus gallus*) breeds, Breast muscle, Liver, Microarray, Metabolic differences, Biological mechanisms

## Abstract

**Background:**

Intensive selection has resulted in increased growth rates and muscularity in broiler chickens, in addition to adverse effects, including delayed organ development, sudden death syndrome, and altered metabolic rates. The biological mechanisms underlying selection responses remain largely unknown. Non-artificially-selected indigenous Chinese chicken breeds display a wide variety of phenotypes, including differential growth rate, body weight, and muscularity. The Wuding chicken breed is a fast growing large chicken breed, and the Daweishan mini chicken breed is a slow growing small chicken breed. Together they form an ideal model system to study the biological mechanisms underlying broiler chicken selection responses in a natural system. The objective of this study was to study the biological mechanisms underlying differential phenotypes between the two breeds in muscle and liver tissues, and relate these to the growth rate and body development phenotypes of the two breeds.

**Results:**

The muscle tissue in the Wuding breed showed higher expression of muscle development genes than muscle tissue in the Daweishan chicken breed. This expression was accompanied by higher expression of acute inflammatory response genes in Wuding chicken than in Daweishan chicken. The muscle tissue of the Daweishan mini chicken breed showed higher expression of genes involved in several metabolic mechanisms including endoplasmic reticulum, protein and lipid metabolism, energy metabolism, as well as specific immune traits than in the Wuding chicken. The liver tissue showed fewer differences between the two breeds. Genes displaying higher expression in the Wuding breed than in the Daweishan breed were not associated with a specific gene network or biological mechanism. Genes highly expressed in the Daweishan mini chicken breed compared to the Wuding breed were enriched for protein metabolism, ABC receptors, signal transduction, and IL6-related mechanisms.

**Conclusions:**

We conclude that faster growth rates and larger body size are related to increased expression of genes involved in muscle development and immune response in muscle, while slower growth rates and smaller body size are related to increased general cellular metabolism. The liver of the Daweishan breed displayed increased expression of metabolic genes.

**Electronic supplementary material:**

The online version of this article (doi:10.1186/s12864-017-3845-9) contains supplementary material, which is available to authorized users.

## Background

For decades intensive genetic selection has increased the growth rate of broiler chickens by more than 300% [1–3], improving feed efficiency, increasing breast muscle size, and increasing weight concomitantly [4–6]. These selection-induced body changes were accompanied by a significant increase in metabolic rate mainly attributable to major metabolic organs including liver and muscle, and increased environmental temperature sensitivity [7–9]. Furthermore, a higher death rate due to sudden death syndrome is observed in these broiler chickens during the early growth phase than in slower growing breeds [10]. The internal organs of broiler chickens develop slower, remain smaller in size, and have reduced oxygen supplies than non-broiler chickens, resulting in several metabolic disturbances [11]. However, the biological mechanisms underlying the relationship between increased growth rates and delayed and reduced organ development remain largely unknown. Detailed studies comparing chicken breed naturally differentiating for growth rate could elucidate these biological mechanisms, resulting in development of biomarkers to control both growth rate and organ development simultaneously.

To enable the study of biological mechanisms underlying a trait, a good animal model should have the following features: (1) the animal model should display high variation for the trait of interest, preferably without an extreme selection background for the trait, as selection may induce unwanted and probably unnoticed side effects, and (2) the animal model should display variation in traits related to the trait under investigation. We studied the biological mechanisms underlying growth rate in (broiler) chickens. As a result of increased growth rate, body weight is usually also increased, while organ weights may be reduced. Indigenous Chinese chicken breeds comprise a wide variety of phenotypes, including growth rate and body weight. Most indigenous Chinese breeds have not undergone artificial selection and are used both as layers and broilers. The Wuding chicken breed is a fast growing large size chicken breed, and the Daweishan mini chicken breed is a slow growing small size chicken breed. The average daily gain (ADG) of the Wuding chicken breed is more than five times the ADG of the Daweishan mini chicken. In addition, the adult Wuding chicken body size and weight are double that of the Daweishan mini chicken. Furthermore, the internal organs of the Daweishan mini chicken are relatively larger than the internal organs of the Wuding chicken [12, 13]. Thus, these two breeds may be an ideal model system to study the effects of broiler chicken growth rates in a background without artificial selection.

Transcriptomics offers the possibility to study genome activation, highlighting the biological mechanisms underlying differences in phenotypes [14–16]. Studying the transcriptome of breast muscle and liver will highlight differences in tissue-specific expression profiles in these two breeds. The objective of this study was to investigate differences in biological mechanisms between these two breeds related to differences in growth rate and body weight phenotypes.

## Results

### Body composition and growth pattern differences in Wuding and Daweishan chicken breeds

**Fig. 1:**
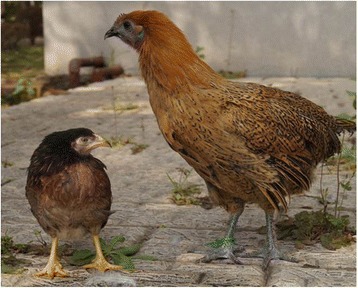
Typical examples of the chicken breeds at the moment of slaughter at 120 days of age: the Wuding chicken (large chicken on the *right*) and the Daweishan chicken (small chicken on the *left*)

**Fig. 2:**
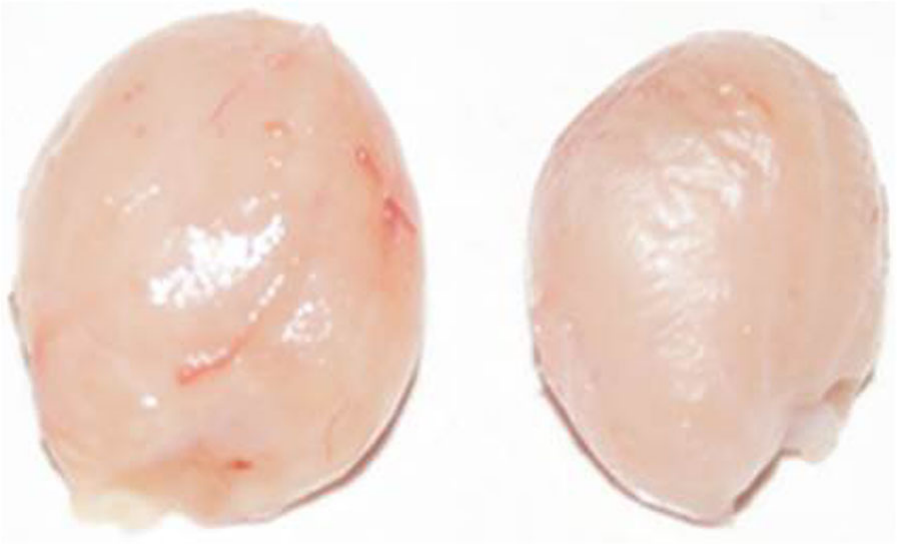
The Bursa of Fabricius of Wuding chicken (on the *left*) and Daweishan chicken (on the *right*). Despite the difference in body size the Bursa of Fabricius were of similar size, so the relative size is larger in the Daweishan compared to the Wuding chicken

Figure 1 shows typical examples of the Wuding chicken (large chicken on the right) and the Daweishan chicken (small chicken on the left) at 120 days of age, the moment of slaughtering, and at which point the animals are still growing. The production characteristics, body weight, and organ weight of the two chicken breeds at slaughter were determined (Table 1). The results showed that the Wuding chicken had a higher daily gain and a higher adult body weight and size than the Daweishan chicken. However, at slaughter the ratio of organ weight to total body weight was higher in the Daweishan chicken than the Wuding chicken. Figure 2 shows the Bursa of Fabricius of Wuding and Daweishan chickens. The Bursa of Fabricius is a major avian immune organ. Despite the difference in body size, the Bursa of Fabricius were of similar size, resulting in a relatively larger size in the Daweishan chicken.

**Table 1:** Organ weights and ratio and percentage of adult body weights, and production characteristics of the Wuding small Daweishan chicken breeds

	Weight (g)		Ratio organ/BW^a^		Organ BW percentage^a,b^	
Organs	Daweishan	Wuding	Daweishan	Wuding	Daweishan	Wuding
Heart	5.61	5.71	0.006	0.003	0.61	0.27
Liver	13.8	18.95	0.015	0.009	1.50	0.90
Lung	4.76	4.79	0.005	0.002	0.52	0.23
Kidney	5.49	5.51	0.006	0.003	0.60	0.26
Spleen	2.27	2.46	0.002	0.001	0.25	0.12
Prescreen	1.91	2.51	0.002	0.001	0.21	0.12
Gizzard	12.88	17.46	0.014	0.008	1.40	0.83
land stomach	3.59	4.06	0.004	0.002	0.39	0.19
Intestine	22.13	24.75	0.024	0.012	2.41	1.18
Trachea	1.22	1.42	0.001	0.0007	0.13	0.07
Gullet	1.64	3.64	0.002	0.002	0.18	0.17
Crop	2.89	4.9	0.003	0.002	0.31	0.23
Bursa of Fabricius	1.17	0.96	0.001	0.0004	0.13	0.05
Thymus	3.58	3.97	0.004	0.002	0.39	0.19
Total average					9.02	4.81
Average daily gain	2.77	14.09				
Feed conversion	12.36	6.07				
Body size	15.66	22.01				
Body weight	920	2100				

^a^ *BW* Body weight; ^b^Organ weight percentage of body weight

### Muscle and liver transcriptome profile differences between Wuding and Daweishan chicken breeds

The (raw) microarray hybridization data for muscle and liver are provided in Additional files 1 and 2, respectively. Table 2 shows the number of differentially expressed genes in the breast muscle and liver of Wuding and Daweishan mini chicken. The number of differentially expressed genes with higher expression in Daweishan than in Wuding chicken was always higher than the number of differentially expressed genes showing the opposite expression ratio.

**Table 2:** Number of genes differentially expressed between the Wuding and the Daweishan chicken breeds, and number of genes with annotation to be analyzed

	N^a^	N analysis^a^
Muscle		
Wuding > Mini^b^	162	139
Mini > Wuding^b^	230	168
All differently expressed genes	392	303
Liver		
Wuding > Mini^b^	45	32
Mini > Wuding^b^	99	73
All differently expressed genes	144	105

^a^N is number of genes ^b^>: Higher expressed

The annotation of the microarray showed many probes as either Expressed Sequence Tag (EST) numbers or unknowns. The probes were converted into gene names, and the number of genes with sufficient identification to be included in the functional analyses is given in the “N analysis” column. Unannotated genes were not used for the functional analyses.

Table 3 shows a real time PCR validation for a number of differentially expressed genes. All genes showed the same directional expression change based on the microarray and real time PCR results, including comparable fold change differences.

**Table 3:** Real time PCR verification of microarray data comparing the expression levels of genes in muscle and liver tissues of Wuding and Daweishan chicken breeds

		Fold change	
Gene symbol	Direction^a^	Microarray	Real time PCR
CATHL2	Wuding > Daweishan	4.14	5.32
GAL7	Wuding > Daweishan	3.95	5.43
GAL4	Wuding > Daweishan	2.48	6.78
GAL1	Wuding > Daweishan	4.17	7.89
CAMP	Wuding > Daweishan	2.31	4.44
MYH6	Daweishan > Wuding	6.18	9.78
TNNI2	Daweishan > Wuding	3.99	7.66
TNNC1	Daweishan > Wuding	3.12	5.45
ACTN2	Daweishan > Wuding	2.33	4.98
MYOM2	Daweishan > Wuding	2.32	5.01

^a^The direction of the differential expression:>: higher expressed

### Genes displaying higher expression in the Wuding than in the Daweishan chicken breed

Breast Muscle (all results in Additional file 3): Cytoscape-ReactomeFI analysis identified one large network and four small networks based on the differentially expressed genes. Three additional analyses methods (DAVID, String, and Cytoscape-ClueGo) indicated that the large network consisted of muscle-specific differentially expressed genes, including muscle developmental genes. Biological mechanisms enriched for differentially expressed genes included both striated skeletal muscle and cardiac muscle development and functioning, as well as mechanisms associated with muscle development and function, including energy metabolism, extra cellular matrix development and composition, protein metabolism, and the scavenger receptor pathway. Another biological mechanism enriched for differentially expressed genes included a network of diverse immune-related functions. Genes involved in the hydroxy lysine and proline metabolisms were also enriched.

**Fig. 3:**
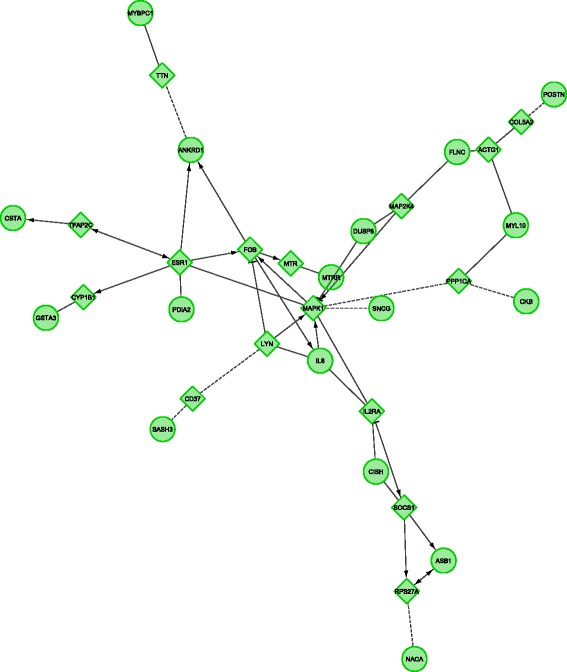
Cytoscape-ReactomeFI network of genes displaying higher expression in Wuding than in Daweishan liver. The network was created using the linker gene option. This software option adds genes to the network through which two or more differentially expressed genes are connected, thereby creating a network. Circles denote differentially expressed genes, diamonds indicate linker genes. Without the use of linker genes no network was created

Liver tissue (all results in Additional file 4): Cytoscape-ReactomeFI could not create a network based on the differentially expressed genes. However, a network was identified when allowing for the addition of linker genes (i.e. genes added by the software linking differentially expressed genes together in a network; Fig. 3). String was unable to create a network, just a few connections between protein could be formed. Functional analysis using DAVID did not identify any enriched biological mechanisms. Cytoscape-ClueGo identified enrichment of genes displaying increased expression involved in catecholamine mechanisms, including dopamine, monoamine, and ammonium ion metabolism.

### Genes displaying higher expressed in the Daweishan than in the Wuding breed

**Fig. 4:**
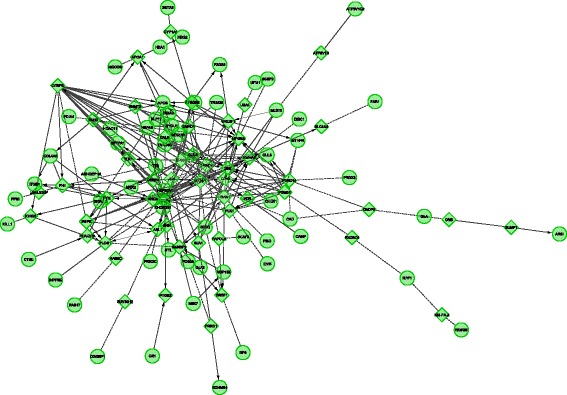
Cytoscape-ReactomeFI network of genes displaying higher expression in Daweishan than in Wuding breast muscle. The network was created using the linker gene option. This software option adds genes to the network through which two or more differentially expressed genes are connected, thereby creating a network. Circles denote differentially expressed genes, diamonds indicate linker genes. Without the use of linker genes no network was created

Breast Muscle (all results in Additional file 5): Cytoscape-ReactomeFI analysis was unable to create a network based on the differentially expressed genes. Allowing the inclusion of linker genes resulted in formation of a network (Fig. 4). As shown in the Figure, no direct gene connections between differently expressed genes were observed. DAVID analysis indicated over representation of genes involved in biological mechanisms of the endoplasmic reticulum. Cytoscape-ClueGo identified enrichment of genes involved in metabolism of glycerolipids/neutral lipids and the scavenger receptor mechanism. String identified networks of differentially expressed molecular chaperones, protein synthesis, T-cell activation, adherence junction, and mitochondrial genes.

Liver tissue (all results in Additional file 6): Cytoscape-ReactomeFI analysis was unable to create a network based on the differentially expressed genes. Allowing for the use of linker genes resulted in formation of a network. Cytoscape-ClueGo indicated overrepresentation of ABC receptors. DAVID and String indicated that genes involved in defense/immune processes – especially the IL-6 cytokine and acute phase mechanisms – and protein metabolism and signal transduction were higher expressed in the Daweishan than in the Wuding breed.

### Biological mechanisms enriched for up- and down-regulated differentially expressed genes (data not shown)

#### Breast Muscle

The results were highly complementary. Only a few additional enriched metabolic mechanisms were identified: the statin pathway and the Selenium-folate-vitB12 (vitamin B12) pathway.

#### Liver tissue

The results identified enriched biological mechanisms associated with the same functions as the individual analysis, namely immune processes and cellular metabolism. Genes involved in immune processes were more commonly differentially expressed than those involved in functional metabolic processes. Enriched functional metabolic processes were primarily involved in the regulation of energy metabolism.

## Discussion

### The model system: breeds, growth rate, body size, and organ development

Breeding in broiler chickens is primarily focused on selection to increase growth rate and muscularity [4–6]. We used two indigenous Chinese chicken breeds to investigate the biological mechanisms underlying differences in body growth rate. High phenotypic variation is observed between these two breeds, with the Wuding breed displaying an almost five times higher growth rate and more than two times higher adult body weight than the Daweishan breed. The high contrast in our model system is ideal for investigating biological mechanisms underlying these phenotypes.

Our results indicated that the Wuding chicken internal organs are relatively smaller than the Daweishan chicken. Increased growth rate in broiler chickens is associated with the development of ascites [11, 17]. Reduced organ development is also observed in chickens with ascites compared to healthy chickens [18, 19]. Ascites has also been described as a metabolic disorder [8, 11], and the liver is the major organ involved in metabolism. We therefore conclude that our model system is ideal for the study of the biological mechanisms underlying phenotypic differences in growth rate, and proceeded to investigate the transcriptome profiles of both breast muscle and liver. Indeed, it may be argued that our model system did not show signs of ascites. The underlying reason may be that the breeds of our model system did not undergo high intensity artificial selection like commercial broiler lines. This resulted in more balanced metabolic changes as we have shown. This may also be a lesson for breeding companies.

### Validation of microarray results using real time PCR

The breast muscle and liver tissue microarrays identified a high number of differentially expressed genes between the Wuding and the Daweishan mini chicken breeds. Genes displaying both higher and lower expression levels were identified between breeds. Microarray results were verified using real time PCR, confirmed both the magnitude and the directional changes reported between the two breeds. Only minor differences in the amplitude of the expression changes were observed between the microarray and real time PCR results. These differences may originate from experimental conditions such as differences in hybridization efficiency between the microarray probes and real time PCR primers, or taq-polymerase efficiencies. Morey et al. [20] showed that the correlation between microarrays and real time PCR varies due to experimental factors including expression level, fold change, etc. Therefore, we concluded that the microarray data used in our bioinformatics analysis was accurate.

### Biological mechanisms: Growth rate and breast muscle development

In both tissues the number of genes displaying increased expression in the Daweishan chicken was higher than in the Wuding chicken breed. This may indicate a higher general metabolic rate in the Daweishan breed than in the Wuding breed. Wuding breed metabolism seems to be focused on specific metabolic pathways, e.g. muscle development (see below), while the general metabolism of the Wuding breed was depressed compared to the Daweishan breed. This may also help explained the relationship between the above-mentioned metabolic effects and ascites.

The Wuding chicken breed displayed increased expression of genes involved in several muscle growth, development, and functional biological mechanisms compared to the Daweishan chicken breed. This result indicates that muscle growth is a major biological factor affecting body growth rates. This is in agreement with previous results from broiler chicken lines selected for increased body growth rate [21], indicating that our model system is a good model for studying the mechanisms underlying differences in growth rates in broiler chickens. Several of our analyses indicated that striated muscle activity and heart functioning pathways were enriched for differentially expressed genes, although we only analyzed breast muscle tissue. This may be due to the involvement of similar genes in striated muscle activity and heart functioning pathways. Furthermore, networks regulating collagen, extra cellular matrix, and energy metabolism accompanied the network. These networks represent parts of breast muscle tissue and necessary muscle functions. This suggests that both muscle growth rate and function are regulated in a balanced way.

The breast muscle results also showed increased expression of genes involved in immune function in the Wuding compared to the Daweishan chicken breed. The results indicated that several immune mechanisms involved in the acute inflammatory reaction were activated (innate immunity and complement system). This may suggest that the Wuding breast muscle tissue was less healthy than the Daweishan chicken. So, despite the balanced biological mechanisms underlying the growth rate of the Wuding breast muscle tissue, the increased inflammatory status may indicate an imbalance compared to the Daweishan chicken.

Next, we investigated the hub genes of the networks in more detail combining the network information with the information from DAVID and Genecards (http://www.genecards.org/). The hub genes were defined as the genes with the most connections to other genes in the networks. This analysis identified several hub genes active in more than one process, indicating a possible relationship between these biological processes.

The FHL2 gene was upregulated in the breast muscle of the Wuding compared to the Daweishan breed. This gene encodes a member of the four-and-a-half-LIM-only protein family, thought to have a role in the assembly of extracellular membranes. This gene is down-regulated during transformation of normal myoblasts to rhabdomyosarcoma cells. Its upregulation in Wuding breast muscle suggests involvement in the regulation of muscle development. The TNNT3 gene was also upregulated in the breast muscle of the Wuding breed. The TNNT3 gene is fast skeletal muscle specific, suggesting that the muscle mass of Wuding chickens is comprised mostly of the fast (white) muscle type. It was previously reported that the muscle fiber composition of high muscle mass chickens consists mostly of fast muscle fibers [22]. However, the upregulation of another hub gene, MYH6, which is mainly involved in heart function, suggests that slow (red) muscle fibers are also responsible for the high muscularity of the Wuding chicken.

The C1QA, C1QB, and C1QC genes are hub genes involved in immune processes (complement and coagulation response, innate immunity, acute inflammatory response, adaptive immune response, and B-cell mediated response) that are also active in the hydroxylysine and hydroxyproline collagen synthesis pathways. COL6A3, another hub gene, is a collagen associated with the C1QA, C1QB, and C1QC genes, the hydroxylysine/hydroxyproline pathways, and muscle organ development. These results suggest that the upregulation of these hub genes is associated with increased growth rates.

The biological mechanisms enriched for genes displaying increased expression in the muscle tissue of the Daweishan compared to Wuding chicken breed were related to more general metabolism. The metabolism of proteins – especially those that need to be exported from the cell – was affected, as was lipid metabolism and molecular chaperones.

The hub genes in the network created after allowing linker genes (i.e. genes added by the software to enable the creation of a network) were either linker genes or differentially expressed genes mainly connected to linker genes. Therefore, we focused on genes central to diverse functions rather than hub genes.

Important genes involved in diverse mechanisms include HSP90B1, HSPA5, PDIA4, and HYOU1. HSP90B1, HSPA5, and HYOU1 are chaperone proteins involved in regulating protein folding. HSP90B1 and HSPA5 are related to endoplasmic reticulum stress. HSP90B1 binds calcium and is ATP metabolizing, while HSPA5 is regulated by glucose levels. Furthermore, HSPA5 is involved in monitoring the transport of proteins through the cell. HYOU1 is also glucose regulated and is also a chaperone protein. Upregulation of HYOU1 is suggested to be involved in reduction of apoptosis. The PDIA4 protein is an electron carrier. Cytoscape ClueGo analysis related these genes, and especially the HSP90B1 gene, directly to the regulation of the subthalamus development, which regulates movement – i.e. skeletal muscle activity. Together these (hub) genes may be involved in regulating increased cellular protein metabolism and breast muscle functioning in Daweishan chickens. Finally, HSP90B1 activity is also associated with immune chaperone activity regulating innate and adaptive immunity [23]. These immune mechanisms differ between the breast muscle of Daweishan and Wuding chicken breeds. Since these genes are involved in regulating the immune response rather than the proinflammatory immune response, these expression changes may not result in increased tissue damage.

### High (muscle) growth rate and ascites in chickens

Although we did not observe ascites in these chicken, health problems related to increased muscle growth rate and body weight of broiler chickens have been reported previously. Specifically, these health problems were related to ascites development and sudden death syndrome [10, 17, 18]. The development of ascites and sudden death syndrome results from an imbalance in the development of the internal organs and skeletal muscle [18, 21]. Indeed we observed differences between the relative organ weights of the Wuding and Daweishan chicken breeds. So, despite the balanced biological mechanisms of the musculature of the Wuding chicken breed, the differential organ development may cause problems leading to increased expression of acute inflammatory genes. Although speculative, this may indicate that the Wuding chicken is less balanced than the Daweishan chicken, and have activated biological mechanisms that could lead to ascites. It should be noted that the growth rate of Wuding chicken is still less than selected broiler lines. We also observed increased expression of the scavenger receptors in the Wuding compared to the Daweishan chicken breed. Scavenger receptors are involved in the removal of foreign substances and waste from tissues [24]. This may further indicate that unhealthy or unbalanced processes exist in Wuding chicken breast. Such processes may potentially be related with inflammation, as described above. They may also be partly responsible for the impaired health of the Wuding chicken.

### Biological mechanisms: Growth rate and liver metabolism

Major differences in liver gene expression levels between the two chicken breeds were not observed, suggesting general liver metabolism is not significantly different between the two breeds. With the use of linker genes a single network was created based on the genes displaying higher expression in the Wuding than in the Daweishan breed, including genes involved in catecholamine related pathways. As all hub genes in the network were linker genes, we will not discuss them further.

The catecholamine pathway is upregulated during high stress levels and also affects the immune response [25–27]. This may help explain the altered expression of genes involved in the immune response (inflammation) in breast muscle tissue. The dopamine and monoamine genes are neurotransmitters that stimulate the reward-motivation behavior and emotion/arousal behavior, respectively. Upregulation of these genes in the Wuding chicken breed may suggest that these chicken are more nervous/agitated compared to the Daweishan breed. In the body dopamine is also involved in gastrointestinal motility and (via norepinephrine mechanism) in regulating vasodilatation. These mechanisms may be necessary to regulate uptake and distribution of feed components required for the higher growth rate of the Wuding chicken breed than for the growth rate of the Daweishan chicken. Vasodilatation may also be a mechanism to overcome the adverse effects of early ascites enabling increased oxygen supply to the cells.

Similarly, in the liver of the Daweishan mini chicken the hub genes in the network created allowing linker genes were either linker genes or differentially expressed genes mainly connected with linker genes. A number of upregulated genes included genes involved in the ATP binding cassette, the ABC receptors, signal transduction, the IL-6 pathway, and a number of transcription factors. The diversity of the identified biological mechanisms indicates higher expression of several metabolic aspects in the Daweishan than in the Wuding chicken breed. As indicated above, these results also indicate that increased growth rates come at the expense of general metabolic activity.

Genes of major importance in this study were the ABC receptor (ATP-binding cassette transporter) genes ABCB1, ABCB11, and ABCC5. All three are involved in multi drug resistance, including Xenobiotic compounds, bile salt export, and cyclic nucleotide export. There main function is to lower the cellular concentration of these substances via translocation. This increases cellular survival. Similarly, the IL6 cytokine functions on cellular proliferation and differentiation to increased cellular survival via the LIF gene and its receptor, all of which showed increased expression in the liver of the Daweishan mini chicken. IL6 is a myokine discharged in the bloodstream after muscle contraction and acts to increase the breakdown of fats and improve insulin resistance. This mechanism represents a direct link between muscle and liver functioning: In Daweishan mini chicken increased muscle cell metabolic activity leads to increased liver activity.

Other important genes include the PSMA1 and the HSPD1 genes. PSMA1 is an ATP/ubiquitin proteasome protein (especially the immunoproteasome) that uses ATP to degrade proteins. This reduces inflammatory immune responses in the liver of Daweishan mini chicken, adding to the increased cellular survival. HSPD1 is a mitochondrial chaperonin protein essential for protein import in the mitochondrion and for correct protein folding. All these functions use ATP, or are involved in ATP synthesis. This points again to the increased metabolic activity in the Daweishan compared to the Wuding chicken.

## Conclusions

Our results identified increased expression of genes involved in muscle development and acute inflammatory responses associated with selection for fast body and muscle growth rates, while the Daweishan breed displayed higher expression of genes involved in general metabolism than the Wuding breed.

The general conclusion of this study is that selection for increased body growth rates focuses on muscle growth leading to a declined and imbalanced general metabolism. This may be the underlying cause of ascites development in broiler chickens. Furthermore, the expression changes in breast muscle seem to be more related to these growth-rate changes than those observed in liver tissue.

## Methods

### Animals, experimental design, and tissue collection

**Fig. 5:**
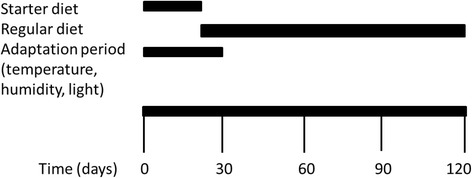
Experimental design. The experiments started with hatched chicken from both breeds (day 0). The chickens were given a starter diet for 21 day, after which the full diet was given. From hatching to day 30 the chickens were slowly adapted to temperature, humidity, and light/dark cycle in an environmentally controlled room

All procedures conducted with the chickens were approved by the Animal Care and Use Committee of the Yunnan Province of P. R. China. One-day-old Wuding and Daweishan chickens [12] were purchased from the Chicken Farm of Yunnan Agricultural University. The same diet was fed to all chickens (Table 4, [28]). Chickens from 1 to 21 days of age (period 1) were fed a starter diet, and older chicken received a full diet (period 2). The chickens had free access to feed and water during the entire rearing period. The chickens were reared in an environmentally controlled room: The temperature was maintained at 35 °C for the first 2 days, and then decreased gradually to 22 °C (45% humidity) until day 30 and thereafter maintained as such to the end of the experiment at day 120; the light was controlled by fluorescent lighting with a light: dark cycle of 12 h each. Figure 5 visualizes the experimental design.

**Table 4:** Diet compositions of the feed of Wuding and Daweishan chicken

Component	Period 1^1^	Period II^2^
Corn	58.85	61.25
Soy protein	25.29	22.44
Wheat bran	8.9	9.5
Fish meal	3	3
Calcium hydrogen phosphate	1.47	1.41
Stone meal	1.1	1
Lysine		0.02
Methionine	0.12	0.11
Salt	0.27	0.27
Minerals and vitamins^3^	1	1
Total nutrients levels	100	100
Metabolism energy (MJ·kg^−1^)	12	12.1
Crude protein	18.5	17
Calcium	0.95	0.95
Phosphorus	0.68	0.65
Lysine	0.96	0.95
Methionine	0.4	0.38

The components of the feed are given in percentage for each component

^1^: Period I is age 1–21 days; ^2^: Period II = older than 21 days of age; ^3^: Supplied per kilogram of diet: antioxidant, 100 mg; biotin, 0.3 mg; vitamin A, 12,000 IU; vitamin D3, 3000 IU; vitamin E, 18.75 mg; vitamin K3, 2.65 mg; vitamin C, 12.6 mg; cyanocobalamin, 0.025 mg; folic acid, 2.2 mg; niacin, 35 mg; pyridoxine, 6 mg; riboflavin, 9 mg; thiamine, 3.0 mg; choline chloride, 600 mg; Co, 0.3 mg; Cu, 12 mg; Fe, 50 mg; I, 1 mg; Mn, 125 mg; Mo, 0.5 mg; Se, 200 lg; Zn, 60 mg

^2^ Diet given to chicken older than 21 days

Six birds per breed were sacrificed at 120 days of age in accordance with the National Experimental Animal Slaughter Standard of China. The birds were selected for similar body weight, 920 g for the Daweishan mini chickens, and 2100 g for the Wuding chickens. Feed was withdrawn 16 h and water 12 h before slaughter. Chickens were weighed immediately before slaughter and sacrificed by cervical dislocation. Breast muscle and liver samples were excised, snap-frozen in liquid nitrogen, and stored at −80 °C. Organ weights were determined immediately after slaughter.

### RNA isolation and microarray hybridization

Tissues were homogenized using a Tissuemiser homogenizer (Fisher Scientific, Houston, TX). Total RNA was isolated using the Trizol extraction method as described by the manufacturer (Invitrogen, Carlsbad, CA). The DNA was removed from the samples using the TURBO DNA free™ Kit (Ambion, Austin, TX) according to the manufacturer’s protocol. The RNA quantity and purity were determined by NanoDrop ND-1000 spectrophotometer at 260/280 nm (Nano Drop Technologies, Wilmington, Delaware). The integrity of total RNA was assessed with an Agilent Bioanalyzer 2100 and RNA 6000 Nano LabChip Kit (Agilent Technologies, Palo Alto, CA). The RNA Integrity Numbers (RINs) for the samples were obtained. Only RNA samples with RIN values of 6 or higher, were used for the analysis.

RNA was pooled per tissue within breeds. Microarray hybridization was carried out by the GeneTech Biotechnology Limited Company (Shanghai, P.R. China) using the Agilent Chicken Gene Microarray (ID: 015068) containing 42,034 probes [29]. The experiments were repeated with dye swapping. Array scanning and data extraction were carried out following standard protocols. The normal distribution of signal plots in every chip was determined using the RMA method. Normalized fluorescence intensity values of each dye-swapped experiment were averaged separately for each sample and reference channels. Thereafter, for each probe, averaged sample and reference fluorescence values were log2-transformed.

The distribution of expressed genes was analyzed by JMP4.0 according to their expression level. If the flag of a gene was “A” by the scanner according to the data normalization and results of Agilent Microarray Suite 4.0 software, it was considered to be not detected, and hence not expressed in this study. Similarly, the genes with “P” flags were considered to be expressed transcripts, and genes with high and low expression levels were defined on the basis of this result. Differential expression was determined using a two-fold difference in gene expression as cutoff value.

### Real time PCR verification of the microarrays

Reverse transcription was performed using 2 μg RNA in a final volume of 25 μL with 10 units of MMLV reverse transcriptase (Promega, Belgium), 1 mM dNTP mixture (Promega, Belgium), 40 units of recombinant RNasin ribonuclease inhibitor (Promega, Belgium) and 0.5 μL of oligo (dT) 18 (Promega, Belgium) in sterilized water and buffer supplied by the manufacturer. After incubation at 42 °C for 60 min, the mixture was heat treated at 95 °C for 5 min. cDNA samples were stored at −20 °C. Real time PCR was performed to verify the microarray quantification of gene expression levels. Each 25 μL PCR mixture contained 12.5 μL of 2 μL iQ™ SYBR Green Supermix, 0.5 μL (10 mmol/L) of each primer (Additional file 7), and 1 μL of cDNA. Determination of the most stable reference genes and their number to be used for normalization of real-time mRNA expression data was done using the geNorm algorithm [30]. The β-actin and 18S rRNA genes were used as the reference genes. The normalization factor was the geometric mean of these 2 reference genes. All primers were designed according to chicken nucleotide sequences in GenBank using Primers Premier 5 and synthesized by Shanghai Shenggong Biological Company (Shanghai, China).

### Bioinformatic analysis

The biological mechanisms associated with growth rate and adult body weight were investigated. The genes differentially expressed between the two breeds in the muscle and liver transcriptome profiles were investigated for over represented biological mechanisms. We investigated the biological mechanisms enriched for genes displaying higher expression in each of the two breeds, and we checked for extra mechanisms using the combined lists of differentially expressed genes.

Many of the microarray probes were poorly annotated. To improve the annotation of the differentially expressed genes the EST numbers were searched in the Metalife database (http://www.metalife.com/Genbank/) and the cDNA sequences were searched using BLAST (Basic Local Alignment Search Tool; http://www.ncbi.nlm.nih.gov/blast). The updated list was used for gene function analysis using DAVID version 6 (The Database for Annotation, Visualization and Integrated Discovery, https://david.ncifcrf.gov/) [31]. The statistical functions of the software were used. Only results significant after correction for multiple testing (*P* < 0.05) using the Benjamini method were reported [32].

Gene network analysis was done with the Cytoscape software using the Reactome FI app [33, 34]. If no network was created the linker gene option was activated. This option is used when no network or an insufficiently small network can be produced using the list of differentially expressed genes. The software option adds genes to the network through which two or more genes from the list of differentially expressed genes are connected, thereby creating a (larger) network. Additionally, the String functional protein analysis tool (http://string-db.org/) was used [35]. This tool not only shows the genes within each network, but also visualizes the genes not within a network. Finally, we used the Cytoscape ClueGo app [36] to visualize networks of physiological pathways over represented within the lists of differentially expressed genes. This app used data from the KEGG and BioCarta databases. First the list of differentially expressed genes was used to identify physiological pathways these genes are active in. The ClueGo app combines the pathways with a common or additive activity into a larger network of pathways [36].

### Differential expression of genes and statistics

The microarray gene expression levels were normalized. If the expression levels of genes differed more than two-fold between the Wuding and Daweishan breeds the genes were considered differentially expressed. All statistics used to identify enriched biological mechanisms and network analyses were provided by the different software packages: DAVID (biological mechanisms), Cytoscape (networks), and ClueGo (networks of pathways). For DAVID analysis we used the Benjamini correction for multiple testing.

## Additional files

Additional file 1: The microarray hybridization data for the breast muscle tissue. The file shows the breast muscle tissue microarray data used for the functional analyses. (XLS 10476 kb)

Additional file 2: The microarray hybridization data for the liver tissue. The file shows the liver tissue microarray data used for the functional analyses. (XLS 10474 kb)

Additional file 3: DAVID, Cytoscape (Reactome-FI and ClueGo), and String analysis for genes displaying increased expression in Wuding compared to Daweishan breast muscle. The file shows all data of the analyses. (XLS 2838 kb)

Additional file 4: DAVID, Cytoscape (Reactome-FI and ClueGo), and String analysis for genes displaying increased expression in Wuding compared to Daweishan liver. The file shows all data of the analyses. (XLS 913 kb)

Additional file 5: DAVID, Cytoscape (Reactome-FI and ClueGo), and String analysis for genes displaying increased expression in Daweishan compared to Wuding breast muscle. The file shows all data of the analyses. (XLS 3250 kb)

Additional file 6: DAVID, Cytoscape (Reactome-FI and ClueGo), and String analysis for genes displaying increased expression in Daweishan compared to Wuding liver. The file shows all data of the analyses. (XLS 1984 kb)

Additional file 7: Real time PCR primers. All primers were designed to anneal at 60 °C. (DOCX 23 kb)

## Abbreviations

ABC receptor: ATP-Binding Cassette transporter (genes ABCB1, ABCB11, and ABCC5)
ADG: Average Daily Gain
BLAST: Basic Local Alignment Search Tool
C1QA, C1QB, C1QC: Complement component 1, Q-subcomponent, A, B, and C chain, respectively)
COL6A3: Collagen type 6, alpha 3
DAVID: The Database for Annotation, Visualization and Integrated Discovery (https://david.ncifcrf.gov//)
EST: Expressed Sequence Tag
FHL2 gene: Four-and-a-Half-LIM-only protein family
HSP90B1: Heat Shock Protein 90 beta 1 (also called Endoplasmin and GRP94),
HSPA5: Heat Shock 70 kDa Protein 5
HSPD1: Heat Shock 60 kDa Protein 1
HYOU1: Hypoxia upregulated protein 1 (HSP70 family member)
IL-6: nterleukin-6
MYH6: Myosin Heavy chain 6
PCR: Polymerase Chain Reaction
PDIA4: Protein Disulfide Isomerase family member 4
PSMA1: Proteasome Subunit Alpha type 1
String: Search Tool for the Retrieval of Interacting Genes/Proteins
TNNT3: Troponin type 3
vitB12: vitamin B12
