# Supplementary material for: Biological mechanisms discriminating growth rate and adult body weight phenotypes in two Chinese indigenous chicken breeds
Source: BMC Genomics. 2017 Jun 20;18:469. doi: 10.1186/s12864-017-3845-9 (PMC5477733; doi:10.1186/s12864-017-3845-9)
Supplement: Supplementary file 7 — Real time PCR primers. All primers were designed to anneal at 60 °C. (DOCX 23 kb) [file 12864_2017_3845_MOESM7_ESM.docx]

Real time PCR primers. All primers were designed to have an annealing temperature of 60^0^C.

| **Accession No.** | **Gene Symbol** | **Primer sequences^1^** | **Product Size (bp)** |
| --- | --- | --- | --- |
| NM_205518 | β-actin | FW: ACGTCTCACTGGATTTCGAGCAGG | 298 |
|  |  | REV: TGCATCCTGTCAGCAATGCCAG |  |
| NM_204305 | GAPDH | FW: GCACGCCATCACTATCTTCC | 356 |
|  |  | REV: CATCCACCGTCTTCTGTGTG |  |
| NM_001001610 | Gal4 | FW: CATCTCAGTGTCGTTTCTCTGC | 321 |
|  |  | REV: ACAATGGTTCCCCAAATCCAAC |  |
| AY621322 | Gal7 | FW: CTGCTGTCTGTCCTCTTTGTGG | 230 |
|  |  | REV: CATTTGGTAGATGCAGGAAGGA |  |
| HQ640432 | CATHL2 | FW: ATCCAGGCTGTGGACTCCTA | 186 |
|  |  | REV: CTCCTTGAAGTCGCAGTCGT |  |
| AY621316 | GAL1 | FW: TTTCCACAGGTGCCAAGGAG | 142 |
|  |  | REV: ATGGGCAAGCAAACATGCAG |  |
| NM_001024830 | CAMP | FW: ACGACTGCGACTTCAAGGAG | 239 |
|  |  | REV: CCTCACGTGGCCCCATTTAT |  |
| NM_001013397 | MYH6 | FW: AGGAGTGCATGTTCCCCAAG | 401 |
|  |  | REV: AAAATGGGGGTGAGTGCTTCT |  |
| NM_205417 | TNNI2 | FW: TGTGGGTGACTGGAGGAAGA | 184 |
|  |  | REV: ATGTTGAGGTTCAGGGCAGG |  |
| NM_205133 | TNNC1 | FW: CAGGCAACTGGAGAGACGAT | 175 |
|  |  | REV: GCACAGAGCAGAAGGGGTTT |  |
| NM_205323 | ACTN2 | FW: CACGCGCTGCTATCTTCAAC | 266 |
|  |  | REV: GTGGGAATTGCACCAAGCTG |  |
| NM_205135 | MYOM2 | FW: CTGAGCTCCAGCGAAAGGAA | 177 |
|  |  | REV: GCGCCTCATCAACGTCAATC |  |

1: FW: forward primer; REV: reverse primer
